# Advances on sonophotocatalysis as a water and wastewater treatment technique: efficiency, challenges and process optimisation

**DOI:** 10.3389/fchem.2023.1252191

**Published:** 2023-08-23

**Authors:** Sivuyisiwe Mapukata, Bulelwa Ntsendwana, Teboho Mokhena, Lucky Sikhwivhilu

**Affiliations:** ^1^ Nanotechnology Innovation Centre (NIC), Advanced Materials Division, Mintek, Johannesburg, South Africa; ^2^ Department of Chemistry, Faculty of Science, Engineering and Agriculture, University of Venda, Thohoyandou, South Africa

**Keywords:** sonophotocatalysis, sonocatalysis, photocatalysis, wastewater treatment, semiconductors

## Abstract

Due to water shortage and increased water pollution, various methods are being explored to improve water quality by treating contaminants. Sonophotocatalysis is a combination of two individual water treatment processes i.e., photocatalysis and sonocatalysis. With advantages including shorter reaction times and enhanced activity, this technique shows possible futuristic applications as an efficient water treatment technology. Herein, background insight on sonophotocalysis as a water and wastewater treatment technique as well as the general mechanism of activity is explained. The commonly used catalysts for sonophotocatalytic applications as well as their synthesis pathways are also briefly discussed. Additionally, the utilisation of sonophotocatalysis for the disinfection of various microbial species as well as treatment of wastewater pollutants including organic (dyes, pharmaceuticals and pesticides) and inorganic species (heavy metals) is deliberated. This review also gives a critical analysis of the efficiency, enhancement strategies as well as challenges and outlooks in this field. It is thus intended to give insight to researchers in the context of facilitating future developments in the field of water treatment, and advancing sonophotocatalysis towards large-scale implementation and commercialization.

## 1 Introduction

Water reclamation and reuse is rapidly gaining attention worldwide due to the heightened water scarcity as a result of industrialisation, climate change and poor resource management (Englande et al., 2015). Access to safe drinking water is therefore becoming an ever-increasing problem in an expanding global economy and increasing population (Boretti and Rosa, 2019). Repercussions of the water shortage include ecosystem degradation, health complications and overall destruction of livelihood (Mishra et al., 2021).

Different techniques have been explored for treating wastewater and enhancing water security, such as flocculation, coagulation, adsorption, ultrafiltration, biodegradation, and reverse osmosis processes (Barakat and Schmidt, 2010; Moghaddam et al., 2010; Park and Hu, 2010; Aneyo et al., 2016; Kryuchkova et al., 2021; Sibiya et al., 2021). However, most of these methods are not cost effective and have several drawbacks such as the inability to achieve total mineralization of the pollutants as well as long treatment times (Al-Tohamy et al., 2022). Additionally, treatment methods like reverse osmosis generate water that is devoid of useful minerals (Sharma and Bhattacharya, 2017).

**FIGURE 1:**
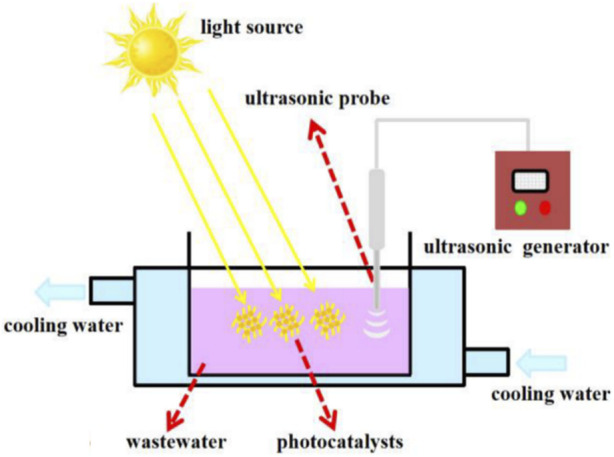
Schematic diagram of the sonophotocatalytic reactor. Reprinted with permission from (Zhang et al., 2021).

Advanced oxidation processes (AOPs) have thus been investigated as efficient methods for the degradation of water pollutants as they rely on the generation of highly reactive radicals which generally mineralise contaminants into CO_2_ and H_2_O (Kaswan and Kaur, 2023). Photocatalysis is generally the most commonly implemented AOP due to its outstanding pollutant degradation capability for a wide range of contaminants (ul Haq et al., 2022). The photocatalysis process is based on the exposure of a photocatalyst to light, leading to the generation of highly reactive oxygen species (ROS), including radicals and peroxides, which break down pollutants and eliminate microorganisms from an aqueous environment (Zhu and Wang, 2017). However, photocatalysis is limited to treating highly transparent wastewater to ensure that the photocatalysts get sufficient light exposure (Wang et al., 2022). Sonocatalytic degradation on the other hand uses ultrasound to generate highly reactive radicals that efficiently degrade a range of pollutants (Eren and Ince, 2010; Wang and Cheng, 2023a). This technology has gained attention because it does not require any pre-treatment of the effluent and it has a stronger penetration ability than light in the catalytic degradation of pollutants (Wang et al., 2017). These two processes can either be implemented separately or concurrently i.e., sonophotocatalysis, which is based on the synergistic interaction of the two individual processes and is therefore a more effective and versatile technique (Wang and Cheng, 2023b). Using a reactor such as that shown in Figure 1 (Zhang et al., 2021), sonophotocatalysis generates clean water upon treatment of wastewater in the presence of a catalyst, which is exposed to both ultrasound and light irradiation.

This review is thus intended to give insight on sonophotocalysis as a water and wastewater treatment technique. Advances that have been achieved in the utilisation of sonophotocatalysis in the disinfection of various microbial species as well as treatment of wastewater pollutants including organic (dyes, pharmaceuticals and pesticides) and inorganic species (heavy metals) are critically analysed. With the aim of contributing to future developments in the field of sonophotocatalytic wastewater treatment, a critical analysis of the efficiency, enhancement strategies as well as challenges and outlooks in this field are also deliberated.

## 2 Sonophotocatalysis mechanism

The mechanisms of activity of sonocatalysis and photocatalysis as well as their synergistic interaction in sonophotocatalysis are detailed below.

### 2.1 Sonocatalysis

**FIGURE 2:**
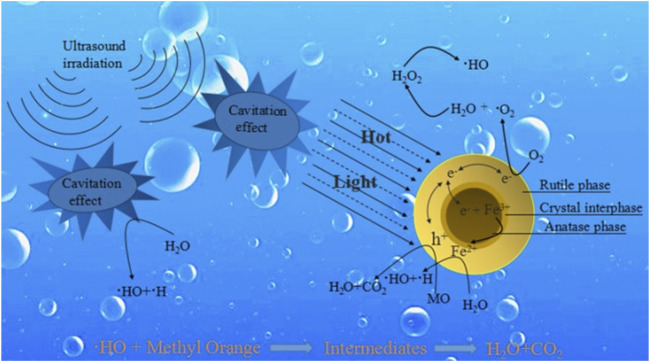
Schematic overview of the sonocatalytic degradation mechanism. Reprinted with permission from (Song et al., 2018).

Sonocatalysis is a process that uses a photoactive material in the presence of ultrasonic irradiation but without the presence of light irradiation. Sonocatalytic degradation technology is highly efficient and operable due to the strong penetration of ultrasound waves in the pollutants during the degradation process (Xu et al., 2023a). Semiconductor materials such as TiO_2_ have been employed for the sonocatalytic degradation of textile wastewater as shown in Figure 2 (Song et al., 2018). This process is largely based on the phenomenon of acoustic cavitation, which is defined as the growth and collapse of pre-existing microbubbles under the influence of an ultrasonic field in liquids. The formation of bubbles is due to the strong decline in local instantaneous pressure induced by strong ultrasound or by some hydrodynamic motion (Mondal et al., 2021; Liu et al., 2023). Under optimum conditions, the bubbles are likely to collapse very violently due to their inherent spherical geometry and the inertia of the surrounding liquid.

At the end of the violent bubble collapse, temperature and pressure inside the bubble significantly increase to more than 4000 K and 300 bar (1 bar = 105 Pa = 0.987 atm), respectively (Yasui, 2021; Xu et al., 2023b). This leads to the formation of reactive species as well as light emission known as sonoluminescence (SL), usually in the presence of a photoactive semiconductor material (Qiu et al., 2018). If the energy of light from sonoluminescence is greater than the band gap of the semiconductor, it causes it to be excited, resulting in the formation of electron-hole pairs (e^–^/h^+^), thereby producing hydroxyl (HO^•^) and superoxide (O_2^•−^_) radicals at the valance and conduction bands, respectively (Thiemann et al., 2017). Additionally, the generated high temperature can possibly provoke the thermal excitation of the semiconductor material, leading to the generation of e^–^/h^+^ pairs by thermal catalysis as well (de Andrade et al., 2021).

The generation of all of these ROS active in sonocatalysis is shown using the equations below:

$$H_{2}O+ultrasound+catalyst\to \;{HO}^{•}+H^{•}$$ (1)

$${HO}^{•}+H^{•}\;\to \;H_{2}O_{2}$$ (2)

$$H^{•}+O_{2}\;\to \;{HO}_{2}^{•}$$ (3)

$${HO}^{•}{+}^{•}OH\;\to \;H_{2}O_{2}$$ (4)

$${HO}_{2}^{•}+{HO}_{2}^{•}\;\to \;H_{2}O_{2}+O_{2}$$ (5)

$$H_{2}O{+}^{•}OH\;\to \;H_{2}O_{2}+H^{•}$$ (6)

The formation of these highly reactive species has allowed the use of sonocatalytic processes in highly contaminated water. Thus, the process is efficient to treat wastewater or effluents containing an array of organic pollutants amongst others.

### 2.2 Photocatalysis

**FIGURE 3:**
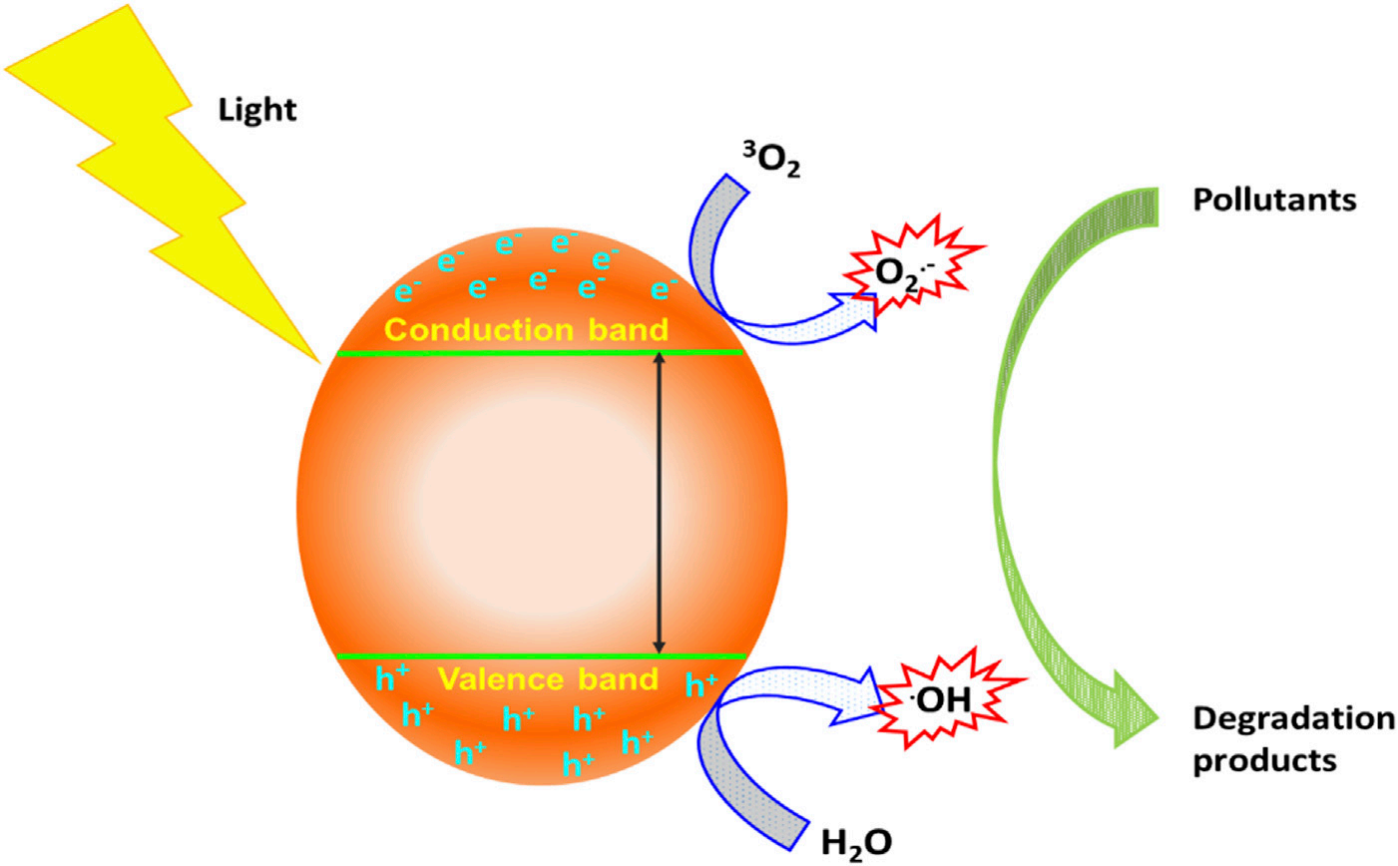
Schematic overview of a semiconductor-mediated photocatalytic treatment process.

The photocatalysis process generally involves reduction and oxidation responses on the surface of photocatalyst material (Tahir et al., 2021). A schematic overview of photocatalytic degradation process is depicted in Figure 3.

Briefly, the absorption of light (photon energy higher than the band gap of the photocatalyst) by a photocatalyst creates holes (h^+^) on the valence band and electrons (e^−^) on the conduction band (Navidpour et al., 2023). The photo-created e^–^/h^+^ pairs mediate the formation of species, like HO^•^ and O_2_^•−^ from atmospheric oxygen and moisture (Fotiou et al., 2014). These species have been reported to have the potential to oxidize and break down organic pollutants, poisonous gas, and eliminate microorganisms from an aqueous environment (Zhao et al., 2020; Xu, 2021). Additionally, the photocatalytic process can also generate hydroperoxyl (HO_2_^•^) radicals which can aid the degradation of inorganic compounds present in industrial wastewater.

The generation of all of these ROS is shown on the equations below:

$$Semiconductor+h\upsilon \;\to \;Semiconductor\;\left(h_{VB}^{+}+e_{CB}^{-}\right)$$ (7)

$$h_{VB}^{+}+e_{CB}^{-}\;\to \;heat$$ (8)

$${OH}^{-}+h_{VB}^{+}\;\to \;{HO}^{•}$$ (9)

$$H_{2}O+h_{VB}^{+}\;\to \;{HO}^{•}+H^{+}$$ (10)

$${HO}^{•}+{HO}^{•}\to \;H_{2}O_{2}$$ (11)

$$e_{CB}^{-}+H^{+}+O_{2}\;\to^{•}{HO}_{2}$$ (12)

H•O2+H•O2 → O2+H2O2 (13)

$$O_{2}+e_{CB}^{-}\;\to \;O_{2}^{•-}$$ (14)

O2•−+H++H•O2 → O2+H2O2 (15)

$$H_{2}O_{2}+e_{CB}^{-}\;\to \;{HO}^{•}+{OH}^{-}$$ (16)

H2O2+HO•→ H2O+H•O2 (17)

The high recombination rate of e^–^/h^+^ pairs in the photocatalyst, as well as agglomeration result in loss of photocatalytic efficiency (Hayati et al., 2020). The combination of photocatalysis with sonocatalysis, however deals with these hindrances and results in highly efficient water treatment systems, hence, sonophotocatalysis is a more efficient water treatment technique (Wang and Cheng, 2023b).

### 2.3 Sonophotocatalysis

**FIGURE 4:**
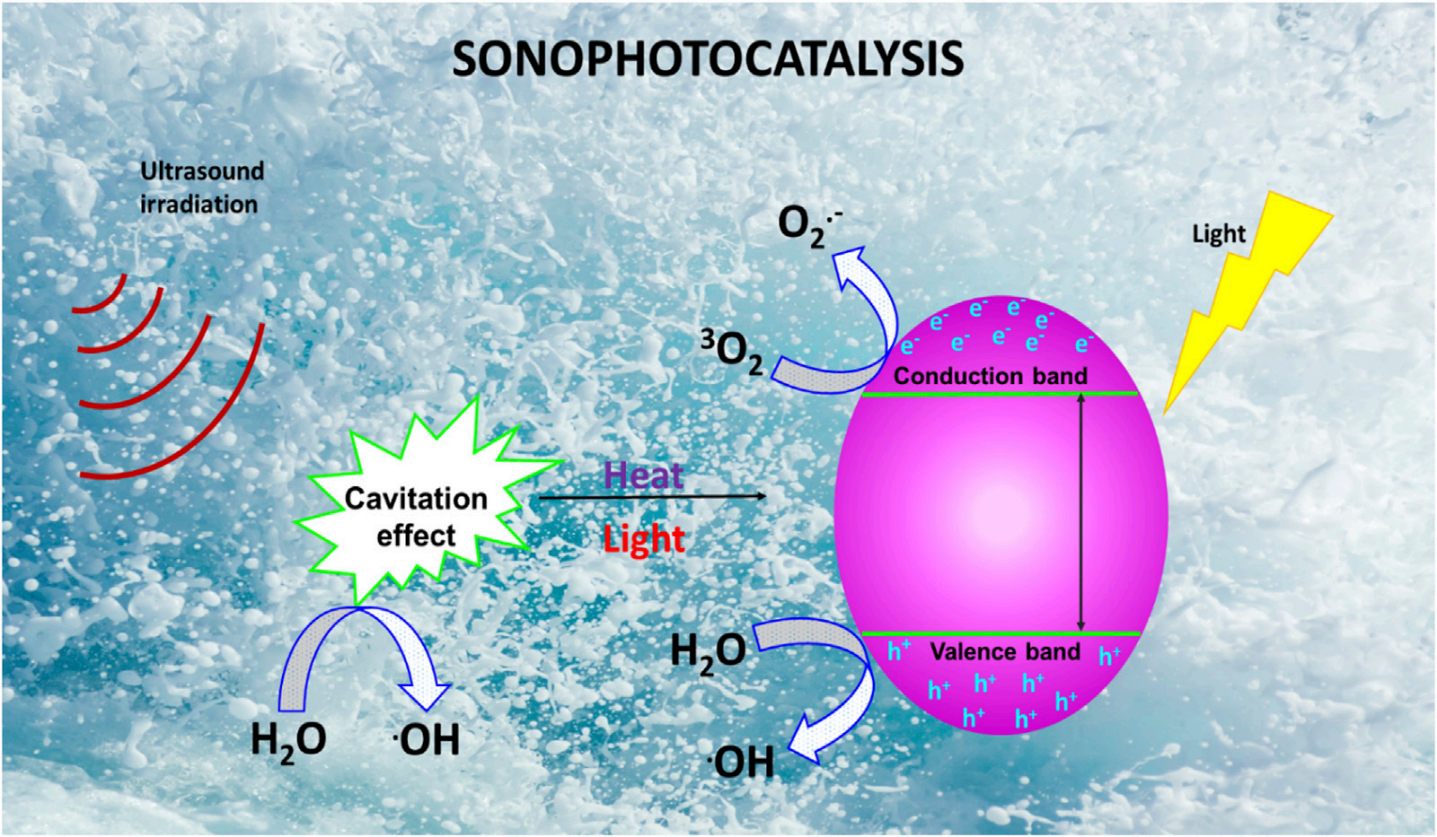
Schematic overview of a sonophotocatalytic treatment process.

Sonophotocatalysis generally entails the combination of light, ultrasound and a catalyst that work synergistically to accelerate the production of ROS, which are highly destructive against water pollutants (Malika and Sonawane, 2022). A schematic overview of sonophotocatalytic degradation process is depicted in Figure 4. Briefly, upon irradiation of a catalyst with ultrasonic waves, sonoluminescence results due to cavitation in the aqueous solution, causing pyrolysis of water molecules and generating highly reactive radicals (Wang and Cheng, 2023b). Additionally, light generated from sonoluminescence can excite the catalyst, leading to the formation of e^–^/h^+^ pairs and subsequently ROS generation (de Andrade et al., 2021). Irradiation of the catalyst’s surface with light increases the generation of ROS as the h^+^ in the valence band react with water molecules adsorbed on the catalyst surface to generate HO^•^, while the e^–^generated in the conduction band react with dissolved oxygen to generate O_2_^•−^, HO^•^, and H_2_O_2_ (Hanifehpour and Joo, 2018). These active species react with pollutants to generate different degradation intermediates and even mineralization products (H_2_O and CO_2_).

Therefore, while the use of light facilitates the degradation of pollutants through the generation of photo-induced ROS, ultrasonication enhance this process by stimulating the production of HO^•^ and assisting in reducing the recombination of photogenerated e^–^/h^+^ pairs (Torres et al., 2008). Ultrasonication also helps with de-aggregation of the catalyst, which enhances its surface area and promotes mass transfer of pollutants between the liquid phase and the catalyst surface (Panda and Manickam, 2017). Moreover, combination of sono- and photocatalysis allows for efficient degradation of both hydrophobic and hydrophilic organic pollutants (Al-Musawi et al., 2021).

In addition to the irradiation sources (light and ultrasound), the overall efficiency of a sonophotocatalytic process also depends on the type of material (sonophotocatalysts) and their efficiency in generating sufficient ROS. Therefore, suitable sonophotocatalysts must exhibit the ability to respond to light and ultrasound irradiation while maintaining good chemical and photo-stability, good electronic properties and efficiency, as well as negligible toxicity and low cost. Semiconductor materials are commonly implemented due to their vital role in lowering the energy barrier for the formation of cavitation bubbles during the sonocatalysis process, while their light-harvesting capacity reveals the population of photogenerated e^–^/h^+^ pairs during the photocatalysis process (Wang and Cheng, 2023b).

Selection of these semiconductors depends on their band gap energies (the minimum energy that is required to excite an e^−^ into the conduction band) which measures photoactivation potentials as shown in Table 1 (Dette et al., 2014; D’Amico et al., 2017; Djurišić et al., 2012; Doyan et al., 2021; González-Borrero et al., 2010; Przeździecka et al., 2021; Xu et al., 2018; Badar et al., 2012; Dalhat et al., 2022; Nurmalasari et al., 2020). Since most of them have large band gap energies (>2 eV), they are mainly activated using UV light. This does limit their real life applications as the UV regime is only a small fraction of the Sun’s energy (<10%) (Wang et al., 2016).

**TABLE 1:** Commonly used semiconductor materials and their respective band gap energies.

Semiconductor	Band gap energies (eV)	Ref
TiO_2_ (anatase)	3.20	Dette et al. (2014)
ZnS	3.60	D’Amico et al. (2017)
ZnO	3.37	Djurišić et al. (2012)
SnO_2_	3.60	Doyan et al. (2021)
WO_3_	2.60–3.00	González-Borrero et al. (2010)
CdO	2.20	Przeździecka et al. (2021)
CuO	1.2–2.6	Xu et al. (2018)
MgO	5.0–7.8	Badar et al. (2012)
CdS	2.42	Dalhat et al. (2022)
Bi_2_O_3_	3.34	Nurmalasari et al. (2020)

Various methods have been employed for the fabrication of these semiconductor sonophotocatalysts, some of the most common being; co-precipitation (El-Sawy et al., 2022), hydrothermal (Kumaresan et al., 2020), sol-gel (Leroy et al., 2020; Norabadi et al., 2020) sonochemical (Anandan and Ashokkumar, 2009) and the solvothermal (Rashid et al., 2023) method. The focus of this review is to showcase the advances that have been made in the application of sonophotocatalysis in water and wastewater treatment, thus these synthesis methods are not detailed.

## 3 Water and wastewater treatment applications

A considerable amount of research has gone into the development of sustainable water treatment techniques and technologies capable of improving the quality of water. The inaccessibility of drinkable water is a critical issue, especially in regions where conventional drinking water treatment systems fail to eradicate toxic waste consisting of aquatic pathogens, metal ions and industrial waste (Ahmed and Haider, 2018). The use of sonophotocatalysis as a possible water and wastewater treatment technique for the most common of these pollutants i.e., organics, microbes and metals has been extensively studied and explored as discussed next.

### 3.1 Degradation of persistent organic pollutants

Due to increased industrialisation and mass production of various products, countless toxic organic chemicals are readily discharged into water bodies, including pharmaceuticals and personal care products (PPCPs), dyes as well as pesticides (Rauf and Ashraf, 2009; Awfa et al., 2018; Fang et al., 2018). Polluted water with refractory organics is difficult to recycle and reuse (Zhang et al., 2020). In addition to sonophotocatalysis, several other treatment technologies [e.g., biodegradation, catalysis, coagulation and adsorption (Patsoura et al., 2007; Le Borgne et al., 2008; Wang et al., 2020; Xiao et al., 2021)] have been applied in the treatment of these hazardous pollutants. However, due to its high treatment efficiency, advances on the use of sonophotocatalysis as a treatment technique for these different organic pollutants is elaborated below.

#### 3.1.1 Neutral, cationic and anionic dyes

Textile industries are the major contributors to water and general environmental pollution as they release undesirable dye effluents (Yaseen and Scholz, 2019). Conventional wastewater treatment methods (physical, chemical and biological) demonstrate several limitations in the elimination of dyes, including low removal efficiencies towards non-biodegradable and refractory organic dyes as well as lengthy treatment times (Crini and Lichtfouse, 2019). These traditional methods are also not very destructive, and usually work by changing the dyes to another form and therefore cause the formation of secondary pollutants. A plethora of research has thus been conducted on the use of sonophotocatalysis as an alternative treatment method for wastewaters containing dyes as listed in Table 2 (Vinu and Madras, 2009; Paramarta and Saleh, 2018; Lops et al., 2019; Razaghi et al., 2021; Al-Hawary et al., 2023; Kucukcongar et al., 2023).

**TABLE 2:** Common organic pollutants treated using sonophotocatalysis and their respective treatment materials and conditions.

Type of organic pollutant	Pollutant name	Material	Preparation method	Catalyst loading (gL^-1^)	Treatment conditions		Treatment efficiency (%)	Ref
					Ultrasound power (W)	Light		
Dyes	Acid Red 14	Fe_3_O_4_@SiO_2_/PAEDTC@MIL-101 (Fe)	—	0.5	36.0	36.0 W	100	Al-Hawary et al. (2023)
	Rhodamine B	ZnO	Sol-gel	0.5	1.00	150 W/m^2^	100	Lops et al. (2019)
	Methylene Blue	Fe_3_O_4_/SnO_2_/NGP	Sol-gel and Co-precipitation	0.3	—	40.0 W	100	Paramarta and Saleh (2018)
	Orange G, Remazol Brilliant Blue R, Alizarin Red S, Methyl Blue, and Indigo Carmine	TiO_2_	Solution combustion	1.0	36.0	80.0 W	—	Vinu and Madras (2009)
	Reactive Red 195	Ag/TiO_2_ and Ag/TiO_2_/Fe_3_O_4_	Co-precipitation	0.1	—	27 W	96 (UVA) 98 (visible)	Kucukcongar et al. (2023)
	Rhodamine B	CuFe_2_F_8_(H_2_O)_2_ and Au/CuFe_2_F_8_(H_2_O)_2_	Solvothermal	0.02	130	—	82.22	Razaghi et al. (2021)
Pharmaceuticals	Tetracycline	Ca doped ZnO	Sol-gel	0.5	100	1.6 W	100	Bembibre et al. (2022)
	Ofloxacin	TiO_2_	Commercial	1.0	8.4	3.16 W/m^2^	98.3	Hapeshi et al. (2013)
	Tetracycline and Ciprofloxacin	Cu_2_O/MoS_2_/rGO	Microwave and Hummer	0.3	120	150 W	100 and 94.0	Selvamani et al. (2021)
	Tetracycline	Au/B-TiO_2_/rGO	Hydrothermal	0.25	600	300 W	100	Vinesh et al. (2019)
	Acetaminophen and Amoxicillin	Mn-doped TiO_2_	Ultrasound	0.10	500	160 W/m^2^	26 and 53	Khani et al. (2019)
Pesticides	Diazinon	Fe doped TiO_2_	Hydrothermal	0.4	100	32.4 MW/cm^2^	85.0	Tabasideh et al. (2017)
	Benomyl	TiO_2_	Commercial	1–3	190	6.53 mW/cm^2^	—	Park (2009)
	Flonicamid	CuO, ZnO and TiO_2_	Commercial	CuO – 1.0 ZnO - 0.75 TiO_2_ - 0.75	100	125 W	CuO – 86.89	Ayare and Gogate (2020)
							ZnO – 91.53	
							TiO_2_ – 98.36	
	Isoproturon	TiO_2_	Commercial	0.1	50	160 W/m^2^	100	Schieppati et al. (2019)

**FIGURE 5:**
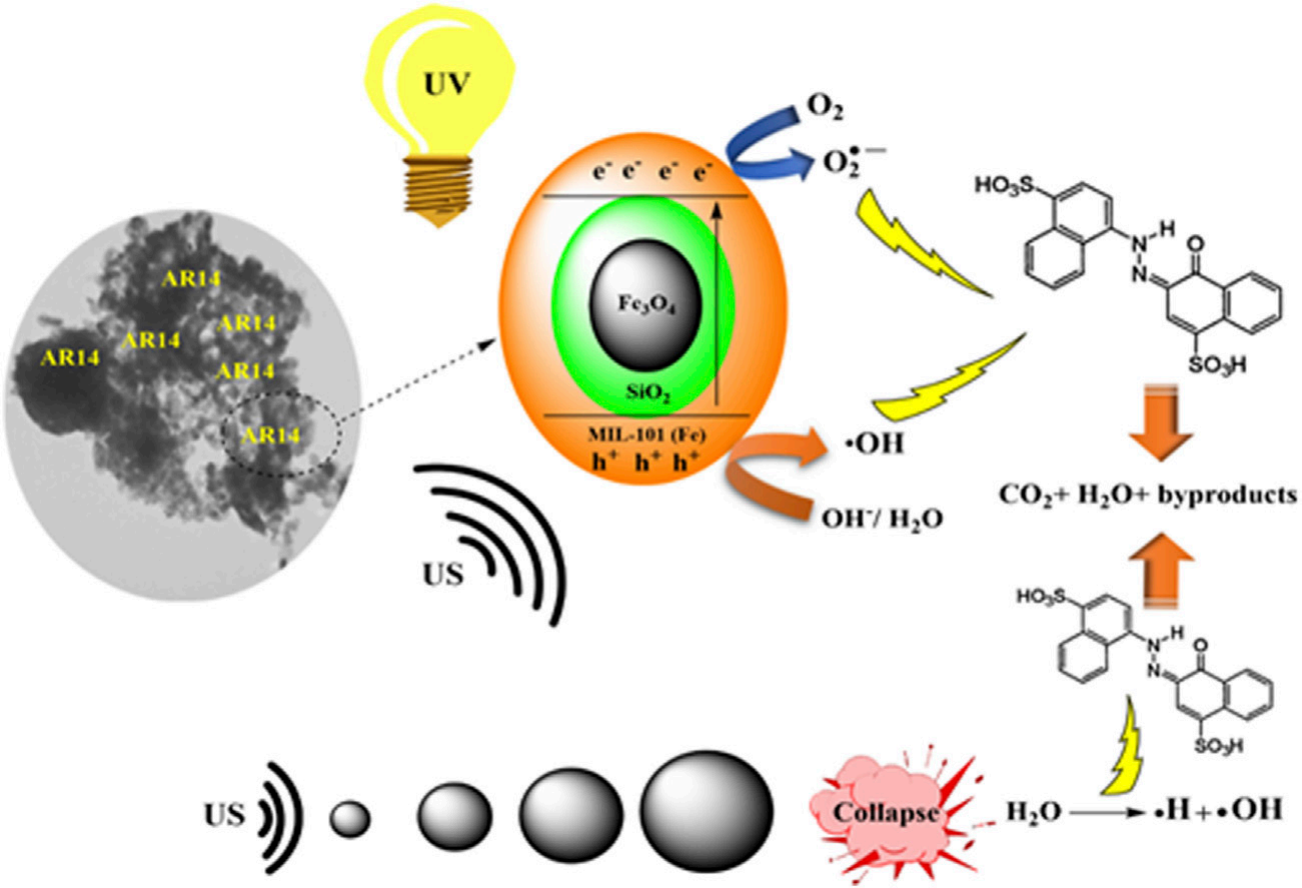
A schematic illustration of decomposition of AR14 under the sonophotocatalysis process. Reprinted with permission from (Al-Hawary et al., 2023).

Al-Hawary et al. (2023) depicted the mechanism of sonophotocatalytic treatment of dyes, using acid red 14 (AR14) as a model pollutant as shown in Figure 5. They reported that the AR14 dye removal can be achieved in solution and catalyst surface through sonolysis and sonocatalytic processes. They also reported that the combination of ultrasound and light radiation was highly efficient as it enhanced the formation of e^–^/h^+^ pairs in the valence and conduction bands of particles resulting in increased ROS generation. Their research findings also showed that the removal efficiency of AR14 increased with increasing operating parameters such as nanoparticle content, ultrasound frequency, and radiation power, while it decreased with increasing the initial pH and initial concentration of AR14 (Al-Hawary et al., 2023).

Just as with photocatalysis, the degradation efficiency in the sonophotocatalysis process can be influenced by the morphology of materials used, since photosensitization occurs on the surface of the semiconductor. To prove this, Lops et al. (2019) synthesized five zinc oxide catalyst powders with different morphologies and sizes i.e., Desert Roses (DRs), Multipods (MPs), Microwires (MWs), Nanoparticles (NPs) and Nanowires (NWs) for the sonophotocatalytic treatment of the cationic Rhodamine B (RhB) dye. They found that the sonophotocatalytic degradation in the presence of DRs microparticles showed the greatest efficiency. The synthesized materials also demonstrated a good stability over repeated cycles of dye treatment (Lops et al., 2019).

Researchers including Paramarta and Saleh (2023) evaluated the efficiency of the sonophotocatalytic degradation. They used Fe_3_O_4_/SnO_2_ composite, deposited onto nanographene platelets (NGPs) using co-precipitation and ultrasound assisted methods. The catalyst reusability tests revealed that the Fe_3_O_4_/SnO_2_/NGP (10wt%) composite could be repeatedly used up to four times without any significant change in the sonocatalytic and sonophotocatalytic activity using cationic Methylene Blue dye as a model pollutant (Paramarta and Saleh, 2018).

Vinu and Madras (2009) studied the sonophotocatalytic degradation of various anionic dyes (Orange G, Remazol Brilliant Blue R, Alizarin Red S, Methyl Blue, and Indigo Carmine) using solution combustion synthesized TiO_2_ (CS TiO_2_) and commercial Degussa P-25 TiO_2_ (DP-25). The rate of sonophotocatalytic degradation of all the dyes and the reduction of total organic carbon was higher compared to the individual photo- and sonocatalytic processes (Vinu and Madras, 2009).

Kucukcongar et al. (2023) investigated the removal efficiency of the neutral reactive red 195 (RR195) dye by photocatalytic and sonophotocatalytic processes under UV-A and visible irradiation using Ag/TiO_2_ and Ag/TiO_2_/Fe_3_O_4_ nanocomposites. Removal of RR195 dye under visible irradiation for 120 min using Ag/TiO_2_/Fe_3_O_4_ was determined as the best with 92% and 96% efficiency for photocatalytic and sonophotocatalytic processes, respectively. They also found that the percentage of RR195 removal under UV-A and visible light decreased with the increase in initial dye concentration and pH values and increased with the increase in light power (Kucukcongar et al., 2023).

Lastly, Razaghi et al. (2021) prepared a stable, mixed-anion sonophotocatalyst CuFe_2_F_8_(H_2_O)_2_ oxyfluoride with narrow band-gap based Au-NPs sensitizer and sacrificial electron mediator for degradation of RhB in aqueous media. The study was the first example of stable degradation of an organic dye driven by visible light coupled with ultrasound wave excitation using an oxyfluoride. They also tested the durability and reusability of the samples for five consecutive cycles under illumination by visible light and ultrasound waves. They found that the sample activities decrease slightly after each repeat reaction cycle under the same experimental conditions, attributing it to a reduction in the active sites of the catalysts, after several sonophotocatalytic degradations (Razaghi et al., 2021).

#### 3.1.2 Pharmaceuticals

Pharmaceuticals and personal care products (PPCPs) constitute one of the largest groups of emerging pollutants as they pertain to medications and therapeutic drugs, cosmetics and other personal hygiene products that promote the general health and wellbeing of humans and animals (Reyes et al., 2021). Research efforts around pharmaceutical agents are however more prominent as most of them last long in the aquatic environment due to lipophilicity, so their cytotoxic effects are chronic rather than acute, even at low concentrations (Zare et al., 2022).

Pharmaceutical agents in drinking water predominantly come from two different sources i.e., production processes of the pharmaceutical industry as well as the common use of pharmaceutical compounds resulting in their presence in urban and farm wastewaters (Gadipelly et al., 2014). Antibiotics are one of the most prominent groups of pharmaceuticals as they are commonly used in human infections, veterinary medicine, and agriculture. The presence of antibiotics has thus been detected in surface waters, ground water aquifers, and even in drinking water in a range of nanogram/L to microgram/L (Sodhi et al., 2021). They are generally released into the environment by various pathways, such as the pharmaceutical industry’s wastewater, wastewater treatment plants, hospitals, as well as human and animal elimination (Samal et al., 2022). This issue leads to the generation of antibiotic-resistant genes (ARGs) and antibiotic-resistant bacteria (ARBs), which accelerate the spread of antibiotic resistance, causing a threat to human health and ecological systems (Serwecińska, 2020).

It has been reported that conventional treatments are not capable of efficiently removing pharmaceuticals as they are predominantly water-soluble and neither volatile nor biodegradable (Abdurahman et al., 2021). As listed in Table 2 (Hapeshi et al., 2013; Khani et al., 2019; Vinesh et al., 2019; Karim and Shriwastav, 2021; Selvamani et al., 2021; Bembibre et al., 2022), numerous researchers have thus explored sonophotocatalysis as a possible treatment alternative for the removal of antibiotics in particular, due to their alarming long-term effects.

Karim and Shriwastav (2021) evaluated the efficacy of photocatalytic, sonocatalytic, and sonophotocatalytic oxidation processes for the degradation of amoxicillin using visible light with nitrogen doped TiO_2_ (N–TiO_2_) nanoparticles as the catalyst. At optimal conditions, maximum degradation efficiencies of 27% and 31% were reported for photocatalysis and sonocatalysis, respectively. The combination of ultrasound and visible light in the presence of N–TiO_2_ enhanced the degradation of amoxicillin due to the reduced bandgap of the catalyst, enhanced cavitation effect, sonoluminescence phenomenon, and improved mass transfer of pollutants, consequently a higher degradation efficiency of 37% was reported for sonophotocatalysis (Karim and Shriwastav, 2021).

Bembibre et al. (2022) investigated the sonophotocatalytic mineralization of tetracycline-based antibiotics using Ca-doped ZnO under LED visible light irradiation. The effects of pH, Ca doping, light and ultrasound intensity were systematically investigated. The ZnO-based catalyst with 2 at% of Ca dopant exhibited the best sonophotocatalytic performance in mineralizing tetracyclines with excellent reusability and minimal sonophotocorrosion i.e., ≤ 1% of catalyst (Bembibre et al., 2022).

Hapeshi et al. (2013) studied the extent of the degradation of ofloxacin in secondary treated effluents by means of sonolysis, photocatalysis, sonocatalysis and sonophotocatalysis using TiO_2_. Various parameters affecting the treatment efficiency including TiO_2_ loading, solution pH, sparging gas, addition of H_2_O_2_, and ultrasound acoustic power were investigated. Sonophotocatalysis was generally faster than the respective individual processes presumably due to the enhanced formation of reactive radicals. It was found that an increase in acoustic intensity was proportional to an increased efficiency of the sonophotocatalytic degradation of ofloxacin. Twenty transformation products were then proposed as a result of the sonophotocatalytic process (Hapeshi et al., 2013).

Selvamani et al. (2021) prepared a ternary Cu_2_O/MoS_2_/rGO composite, synthesized by the microwave method, for the sonophotocatalytic degradation of tetracycline and ciprofloxacin. The composite demonstrated a synergic sonophotocatalytic degradation of tetracycline (20 mg/L) and ciprofloxacin (10 mg/L) antibiotics with high efficiency of 100% and 94% within a short duration of 10 and 75 min, respectively. They further elucidated that the synergic effect of degradation is due to the spontaneous production of HO^•^ and O_2_^•−^ radicals favouring free e^−^ to actively participate in the degradation process (Selvamani et al., 2021).

Vinesh et al. (2019) reported on the incorporation of e^−^ deficient boron atoms along with Au doped TiO_2_ in the presence of rGO support for the sonophotocatalytic degradation of tetracycline using visible light illumination. They found the individual effect of photocatalysis and sonocatalysis for the degradation of tetracycline to be 45% and 12%, respectively, whereas complete degradation (100%) was achieved with 1.3 folds synergistic effect for sonophotocatalysis in 1 h. The enhanced degradation activity was mainly attributed to combined effect of rapid e^–^/h^+^ pair separation facilitated by e^−^ deficient B-atoms and rGO support and physical forces of ultrasound as well (Vinesh et al., 2019).

**FIGURE 6:**
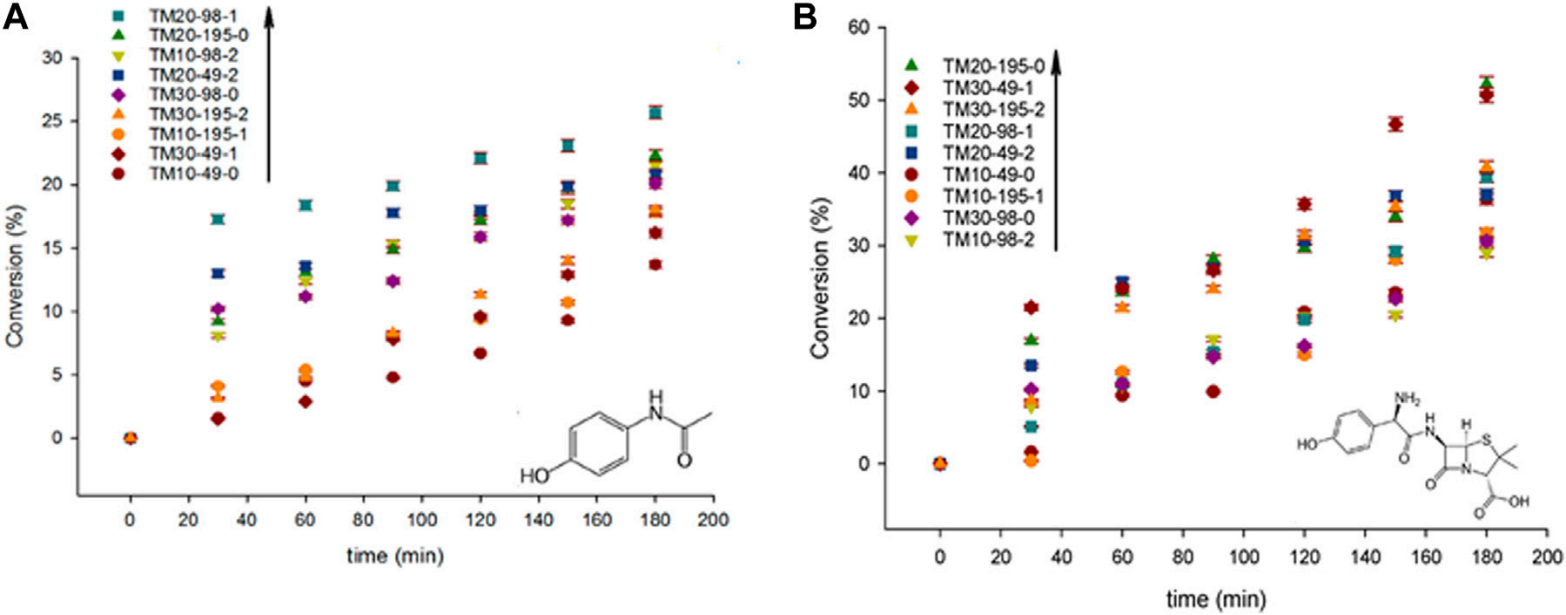
**(A)** acetaminophen and **(B)** amoxicillin degradation with various Mn-doped TiO_2_ catalysts; error within ± 2%. Reprinted with permission from (Khani et al., 2019).

Khani et al. (2019) reported on the synthesis of Mn-doped TiO_2_ by ultrasound for the sonophotocatalytic degradation of acetaminophen and amoxicillin. They found that the ultrasound synthesized samples had a higher brookite content and wider distribution of the band-gaps, in the 1.6–1.91 eV range, while traditional ones ranged from 1.72 to 1.8 eV. The catalysts synthesized with ultrasound were also up to 50% more active than the traditional samples. Interestingly, as shown in Figure 6, their results also showed that amoxicillin decomposed more easily than acetaminophen due to its different molecular properties (pKa and polar surface area). The maximum AMO degradation achieved was 53% with the catalyst with the smallest band-gap (1.6 eV) and the highest surface area (158 m^2^ g^−1^), whereas the maximum APAP degradation was 26% with the catalyst with the band-gap of (1.7 eV) and the surface area of (132 m^2^ g^−1^) (Khani et al., 2019).

#### 3.1.3 Pesticides (herbicides, fungicides and insecticides)

Although pesticides are recognised as reagents for protecting crops against harmful pests, their runoff from agricultural fields into water bodies has detrimental health effects to aquatic organisms and humans (Syafrudin et al., 2021). This is attributed to their ability to interfere with the normal functioning of a large number of species including human beings and animals (Shefali et al., 2021). They can pose severe health risks upon ingestion i.e., they have potential as carcinogenic agents as well as endocrine disruptors (Damalas and Eleftherohorinos, 2011). These pesticides are usually categorized depending on the kind of pest they regulate, i.e., insecticides are used for controlling insects, herbicides for weeds and fungicides are used for controlling fungi. Ideally, they should be toxic to the target organisms but that is not always the case as they leach into water bodies easily. Hence as listed in Table 2 (Park, 2009; Tabasideh et al., 2017; Schieppati et al., 2019; Ayare and Gogate, 2020), numerous researchers have explored sonophotocatalysis as a promising treatment method for the different types of pesticides relative to other treatment methods i.e., photocatalysis and sonocatalysis.

Tabasideh et al. (2017) conducted a comparative study on the sonocatalytic, photocatalytic, and sonophotocatalytic degradation of diazinon (insecticide) using iron-doped TiO_2_ nanoparticles. They found sonophotocatalysis to be a better treatment method for diazinon than sonocatalysis and photocatalysis. Their results also revealed that the degradation of diazinon increased with the increase of the concentration of catalyst and Fe doping concentration in all three cases (sonocatalytic, photocatalytic, and sonophotocatalytic), while the degradation efficiency decreased with the increase of initial diazinon concentration and pH (Tabasideh et al., 2017).

Park (2009) reported on the degradation of benomyl (fungicide) by a sonophotocatalytic and photocatalytic system for comparison. Under the optimal conditions, i.e., initial benomyl concentration was 3.2 mg L^-1^ and the concentration of TiO_2_ was 2 g L^-1^, degradation rates from the sonophotocatalytic system were about 1.5 times higher than those from the photocatalytic system (Park, 2009).

Ayare and Gogate (2020) reported on the efficacy of sonocatalytic, photocatalytic and sonophotocatalytic oxidation of flonicamid (insecticide) solution using various catalysts (CuO, ZnO, and TiO_2_). Sonophotocatalysis treatment was found to be the most effective treatment approach for mineralization of flonicamid solution using the different catalysts. The proposed mechanisms showed enhanced generation of oxidizing species, accelerating the formation of cavities and enhancing the catalytic activity at the catalyst surface (Ayare and Gogate, 2020).

Schieppati et al. (2019) studied the individual, additive and synergistic degradation action of photolysis, sonolysis, sonophotolysis, and sonophotocatalysis against isoproturon (herbicide) by varying catalyst loading and/or ultrasound power for the last three techniques. With 0.1 gL^−1^ catalyst, photocatalysis and sonophotopcatalysis completely degraded isoproturon within 240 and 60 min, respectively (>99% conversion). Sonophotocatalysis was also found to break isoproturon down into smaller molecules than photocatalysis alone (Schieppati et al., 2019).

### 3.2 Heavy metal treatment

The treatment of heavy metals in wastewater has proven to be a challenge that requires attention, as heavy metals are major wastewater pollutants that are not biodegradable, are toxic in the human body and can accumulate in the ecosystem (Vidu et al., 2020). Typical industrial methods employed in wastewater treatment, such as adsorption, chemical precipitation, ion exchange, ozonation and biological methods cannot efficiently reduce the metal concentration in water to within the regulatory standards effectively. This is because there is a considerable number of heavy metals in water that are complexed with organic chelating agents that come from textile, nuclear, and electroplating sources (Gao and Meng, 2021). Research on the sonophotocatalytic treatment of heavy metals is also very limited and is an area worth advancing, as the little work that’s been done has yielded promising results which demonstrate feasibility of this technique.

For instance, Chen et al. (2021) prepared zirconium–porphyrin metal–organic frameworks (MOFs) for the visible-light-driven sonophotocatalytic reduction of toxic Cr(VI) to Cr(III) in water. They found that the catalysts exhibited enhanced activities for Cr(VI) reduction compared with the photocatalytic process. Using fluorescence and UV–vis absorption spectra measurements, they were also able to deduce that the sonophotocatalytic process promotes the transfer of photoinduced electrons from the catalyst to Cr(VI), hence enhancement in the catalytic performance (Chen et al., 2021).

Chen et al. (2022) also prepared titanium–porphyrin MOFs and used them as visible-light-driven catalysts for the sonophotocatalytic reduction of Cr(VI). All the catalysts presented higher efficiency in the reduction of Cr(VI) to Cr(III) in aqueous solution when sonophotocatalytic treatment was employed than in photocatalysis. Sonophotocatalytic experiments and electron paramagnetic resonance measurement proved that the Ti-oxo chain units and porphyrin ligand in the structures of the catalysts existed as catalytic active centers for sonophotocatalytic reduction of Cr(VI). They also reported that photoluminescence and UV absorption spectra revealed that the synergy between photocatalysis and sonocatalysis strengthened the migration of photogenerated e^−^ from the catalyst to Cr(VI), which improved the activities of catalysts (Chen et al., 2022).

### 3.3 Microbial disinfection

The disinfection of water is useful for the elimination of the pathogens that are responsible for waterborne diseases (Liu et al., 2021). Traditional disinfection methods typically entail the use of chlorine or other oxidative chemicals in water. There is however growing concern over the formation of harmful disinfection byproducts during chlorine- or chemical-based disinfection, which has heightened the need to develop alternative processes for water disinfection (Qiu et al., 2020).

**FIGURE 7:**
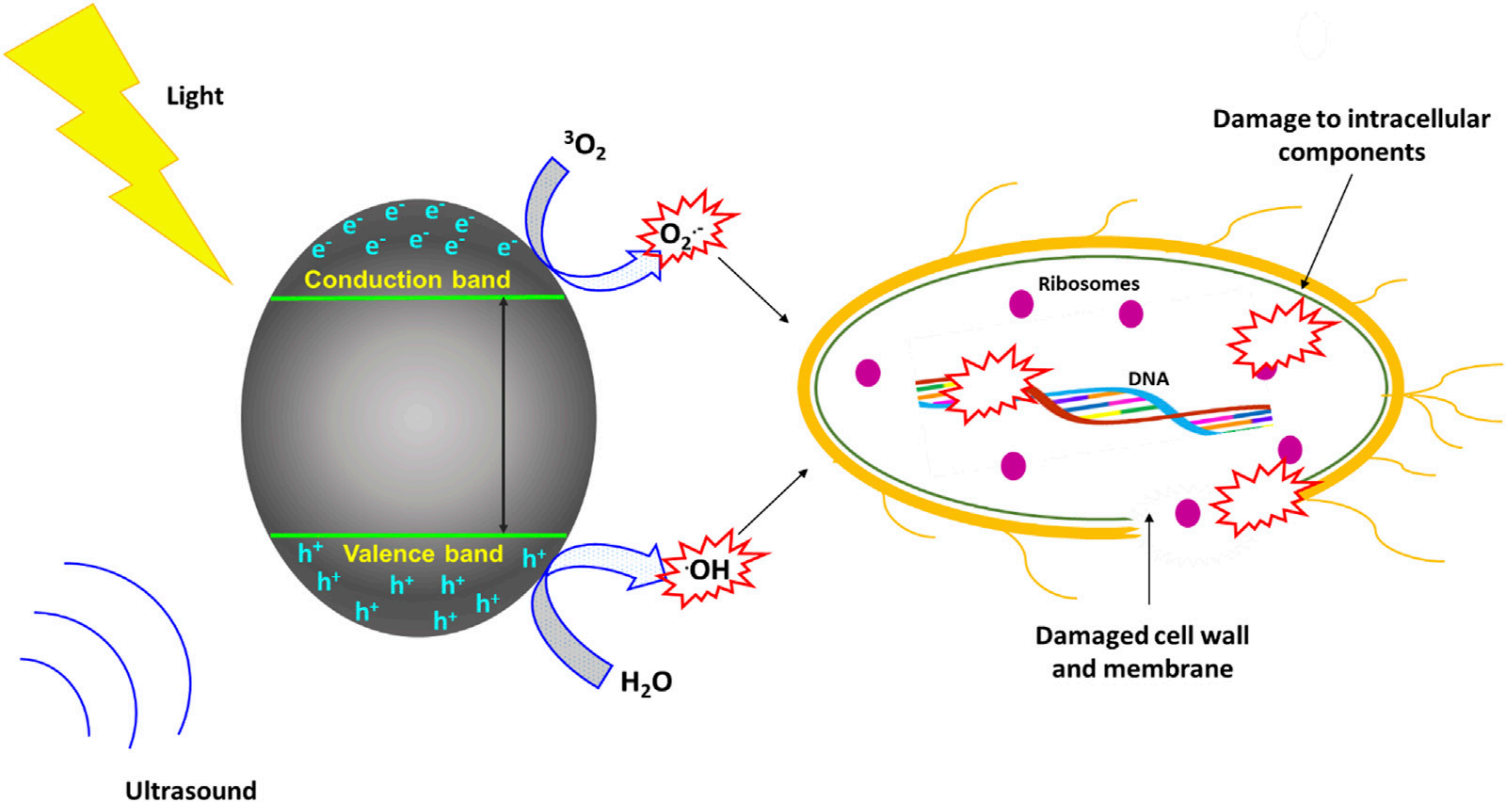
Mechanism of sonophotocatalytic treatment of microbes.

Although still at its infancy stages, sonophotocatalysis has been explored as a viable method for the treatment of microbes, especially bacteria. The combined effects of ultrasonication and photocatalysis in microbes is depicted in Figure 7. The physical or mechanical consequences of ultrasound cavitation (such as shock waves and shear pressures) cause the mechanical rupturing of cell membranes and lead to cellular lysis (Dai et al., 2020). Additionally, the generated ROS from both sonocatalytic and photoinduced chemical reactions result in the damage of DNA and other intercellular components and they are then ejected from the cell due to cytoplasmic material leakage (Moradi et al., 2022).

Rahman et al. (2020) reported on the sonophotocatalytic treatment of *Salmonella Typhimurium* using Fe-doped ZnO nanoparticles (ZnO:Fe) wherein complete disinfection of the targeted bacteria was achieved within 45 min. The efficiency of ZnO:Fe nanoparticles was found to be higher than that of conventional sonophotocatalysts (ZnO and TiO_2_). Their investigations also indicated that the HO^•^ and O_2_^•−^ radicals could have been the key ROS interfering with the oxido-reductase protein system of the bacteria and hence hindered its metabolic activity. Additionally, they reported that the treatment process did not result in any process resistance, making it a good candidate for water disinfection (Rahman et al., 2023).

Mukherjee et al. (2022) reported on the efficiency of sonophotocatalysis in eradicating multi-drug resistant *Klebsiella pneumonia*. Hydrothermally synthesized CdS nanorods were applied as the sonophotocatalysts under blue light irradiation from a LED source in combination with low frequency ultrasonication for the complete disinfection of the coliform bacteria. Various reaction parameters including light intensity, sonication power, and catalyst dose were found to affect the disinfection capability. Following optimisation, the disinfection strategy was able to handle 106 CFU/mL of *K. pneumonia* efficiently in 20 min. The sonophotocatalyst demonstrated good reusability and stability. They elucidated that sonophotocatalysis is a viable disinfection technology which utilises the release of ROS and mechanical shear to induce bacterial disinfection (Mukherjee et al., 2022).

Drosou et al. (2010) reported on the efficacy of heterogeneous photocatalysis and sonophotocatalysis induced by UV-A irradiation and low frequency (24–80 kHz) ultrasound irradiation in the presence of TiO_2_ and peracetic acid (PAA) as an additional disinfectant to inactivate *Escherichia coli* in water. PAA-assisted UV-A/TiO_2_ photocatalysis generally led to nearly complete bacterial inactivation in 10–20 min of contact time with the extent of inactivation depending on the photocatalyst type and loading (in the range 100–500 mg L^−1^) and PAA concentration (in the range 0.5–2 mg L^−1^). PAA-assisted UV-A/TiO_2_ sonophotocatalysis however showed complete bacterial inactivation with shorter treatment times and lower PAA doses and was therefore reported as the most efficient treatment technique (Drosou et al., 2010).

## 4 Efficiency and enhancement strategies

From the reported findings of several researchers, it can be deduced that sonophotocatalysis has greater efficiency in the treatment of water pollutants at different conditions relative to photocatalysis and sonocatalysis. The high ROS generation efficiency in sonophotocatalytic treatment contributes to the versatility of the process i.e., treatment of a range of water pollutants. Additionally, sonophotocatalysis eliminates some of the main disadvantages observed in each individual technique including high costs, sluggish activity, and prolonged reaction times (Moradi et al., 2022). The synergy between the ultrasound and light irradiation moreover causes an enhanced photocatalyst activity due to the ultrasonic separation of photocatalyst particles, which in turn increases the surface area of the photocatalyst for increased exposure to light (Yetim and Tekin, 2017). Furthermore, ultrasound can be used as an irradiation source to induce sonocatalytic activity in the photocatalysts even if light radiation is blocked because of trapping of the photocatalyst in the pores of its support (Joseph et al., 2009). Lastly, ultrasound helps in catalyst cleaning and regeneration (Moradi et al., 2022). This possibly increases the available active sites and thus enhances the light absorption and catalyst reusability efficiency. As discussed next, various strategies have been employed for enhancing the efficiency of sonophotocatalysis, either by modifying the sonophotocatalysts or the sonophotocatalytic process itself.

### 4.1 Sonophotocatalyst modification

One of the prevalent strategies employed for further enhancing the efficiency of sonophocatalytic treatment of water pollutants is the modification of the sonophotocatalysts. Modification of properties such as physical, electronic and magnetic properties in nanometer scale help with enhancement the sonophotocatalytic activity. This can be achieved by various methods including; doping the sonophotocatalysts with metals or non-metals (Tabasideh et al., 2017; Rajoriya et al., 2019). The dopants help increasing the absorption band width into the visible region as well as reducing the hole–electron recombination (Chakma and Moholkar, 2015). This corroborates with Paul et al. (2017) who evaluated the sonophotocatalytic efficiency of pure and Ag doped flower-like hierarchical molybdenum oxide nanorods for the degradation of Methylene Blue dye under diffused sunlight. They found that the optical band gap energy decreased with Ag doping while the degradation efficiency was increased (Paul et al., 2017).

**FIGURE 8:**
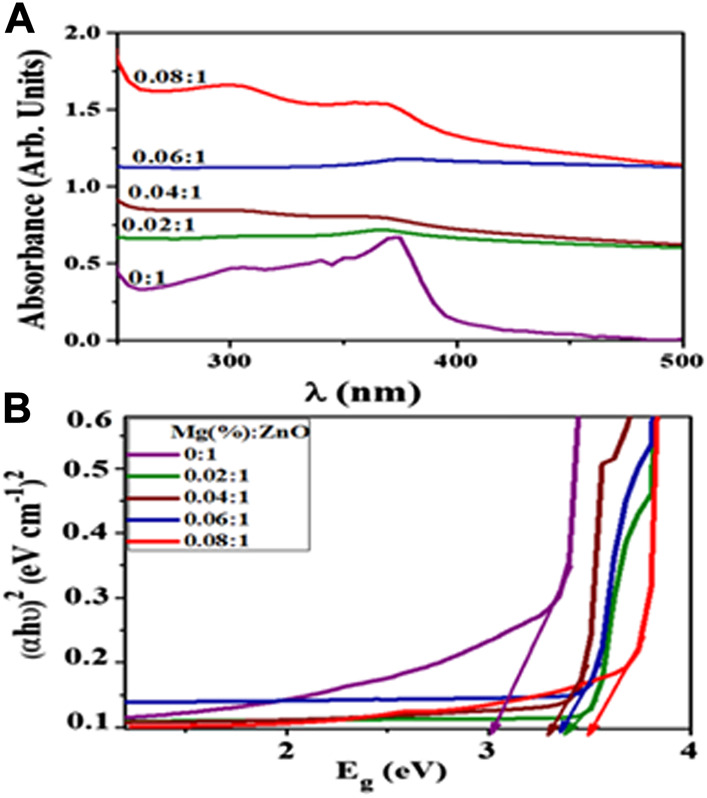
**(A)** Absorption spectra and **(B)** Tauc plots of samples of Mg-doped ZnO. Reprinted with permission from (Ikram et al., 2021).

Anomalies do occur though, for instance, Ikram et al. (2021) prepared various concentrations of Mg-doped ZnO nanorods which they applied in the photocatalytic, sonocatalytic and sonophotocatalytic degradation of a mixture of Methylene Blue and ciprofloxacin. As shown in Figure 8A, they observed a shift in absorption upon doping which they attributed to oxygen deficiency, particle size effect and grain structure defects. They extracted values from Figure 8A to calculate optical band gap (E.g.,) of ZnO (using Tauc plots), which increased from 3.32 to 3.72 eV with increased Mg doping (Figure 8B). They explained the blueshift in E.g., based on the Burstein–Moss effect phenomenon. They explained that in the metal oxide method, particle size reduction can result in a blueshift of the band gap due to the quantum confinement effect (QCE). They further elaborated that doping may also affect local symmetry and generate lattice defect centers that change the structure of the band and induce significant shifts in optical properties. Nonetheless, the experimental results however showed improved degradation performance by Mg-doped ZnO nanorods relative to the bare ones (Ikram et al., 2021).

**FIGURE 9:**
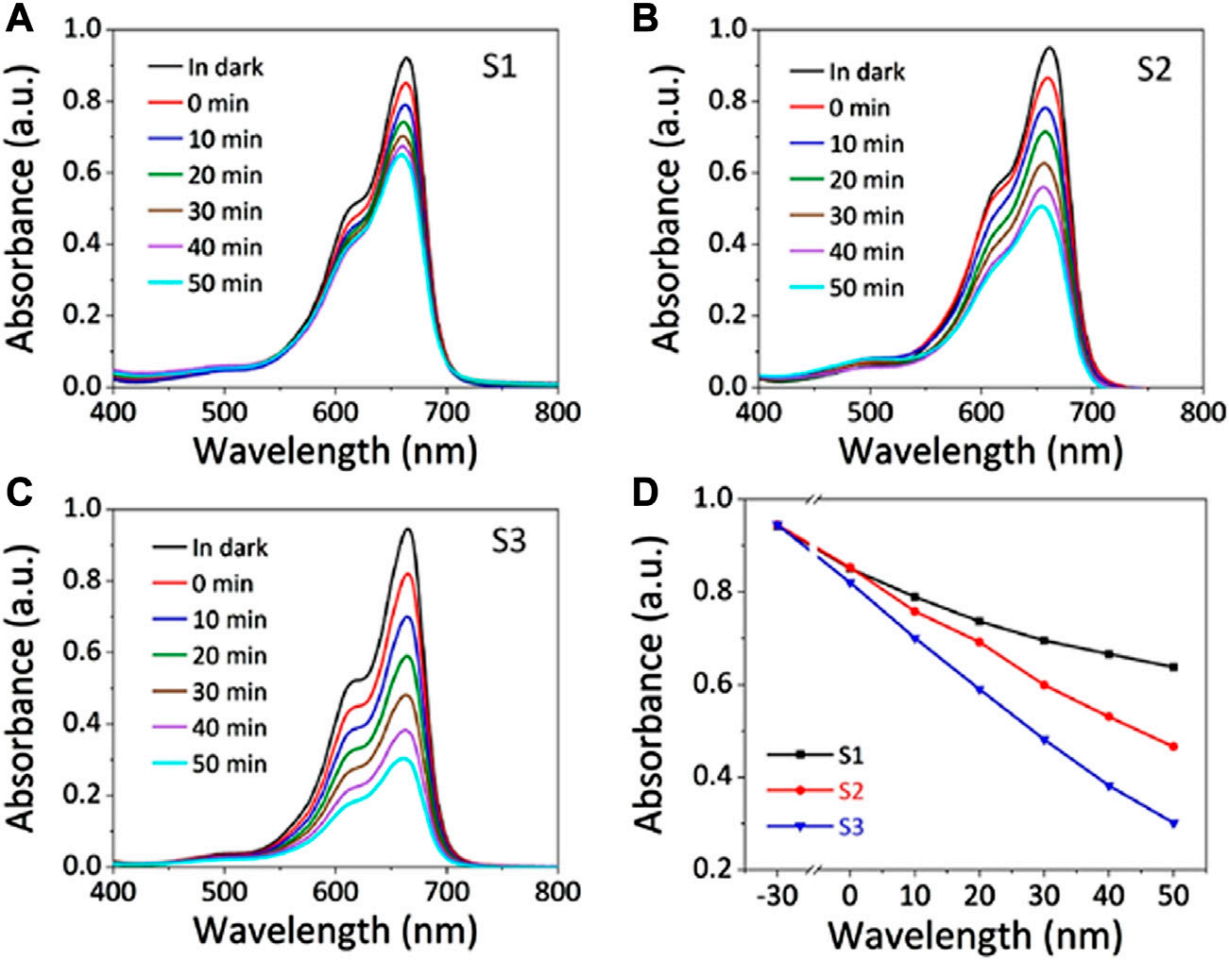
Sonophotocatalytic capabilities of the three samples. **(A–C)** Absorption curves of methylene blue sonophotocatalyzed by **(A)** ZnO nanorods, **(B)** ZnO/ZnS nanorods, and **(C)** ZnO/ZnS nanotubes. **(D)** Sonophotocatalytic degradation rates of the three samples. Reprinted with permission from (Guo et al., 2021).

Another promising strategy for enhancing sonophotocatalysis is by coupling the nanoscale semiconductor sonophotocatalyst with another lower band gap semiconductor i.e., creating heterojunctions (Zhang et al., 2021). The close contact of semiconductors with different Fermi levels leads to the formation of an internal electric field. This electric field can direct the movement of electrons and holes through the interfacial zone (Moradi et al., 2022). For instance, Guo et al. (2021) investigated the influence of the heterojunction by comparing the sonophotocatalytic capabilities of ZnO and ZnO/ZnS core–shell nanorod arrays in the degradation of Methylene Blue. As depicted in Figure 9, they observed that the nanorods with the heterojunction i.e., ZnO/ZnS core–shell had a higher degradation efficiency (45.4%) than ZnO (25.1%). They also studied the morphological influence in the heterojunctions by comparing ZnO/ZnS nanorods and ZnO/ZnS nanotubes which further showed that the latter had a higher capability of 63.3%. They ascribed the improvement to the coupling effect of the enhanced piezoelectric field and the reduced migration distance, which suppresses the recombination of photoexcited e^–^/h^+^ pairs while transforming the morphology from nanorod to nanotube (Guo et al., 2021).

**FIGURE 10:**
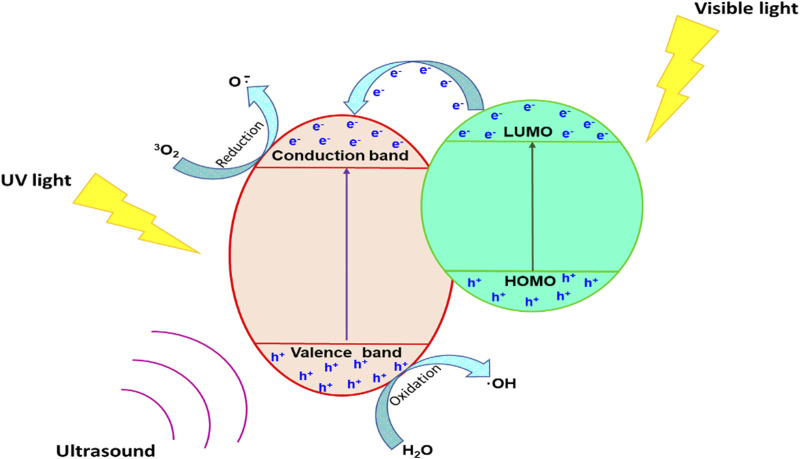
The mechanism of synergistic interaction of dye photosensitised semiconductor sonophotocatalysts.

The sonophotocatalysts can also be modified by dye photosensitisation i.e., conjugation of the semiconductor sonophotocatalyst to a dye (Kaur and Singh, 2007; Mkhondwane et al., 2023a). This results in composite materials with two light absorbers, wherein the dye absorbs visible light, which is beneficial as most of the solar irradiation is in the visible region. The synergistic interaction of a dye with a semiconductor photocatalyst is depicted in Figure 10. The excited dye causes an influx of electrons in the conduction band of the semiconductor, thereby enhancing the generation of ROS which can then decompose a range of pollutants. Moreover, ultrasonic cavitation may cause the pyrolysis of water molecules in contact with the surface of the sonophotocatalyst, thereby further enhancing the generation of ROS (de Andrade et al., 2021).

Mkhondwane et al. (2023) investigated the use of two Zn phthalocyanines as photosensitisers for TiO_2_ fibers in sonocatalytic, photocatalytic, and sonophotocatalytic degradation of Rhodamine 6G. They found that the ZnPc functionalized fibers displayed higher activity than their corresponding bare ZnPc analogous. Additionally, the sonophotocatalytic process displayed higher degradation aptitude than sonocatalytic and photocatalytic processes (Mkhondwane et al., 2023b). Moreover, Hakki and Allahyari (2022) prepared Bi_2_O_3_-ZnO photocatalysts with various amounts of copper phthalocyanine as a photosensitiser for the sonophotocatalytic degradation of Methyl Orange dye. They compared the sonophotocatalytic degradation of Methyl Orange under simulated solar light with sole photocatalysis and sonolysis, and the results showed that the sonophotacatalytic degradation was the most efficient (Hakki and Allahyari, 2022).

### 4.2 Process modification

The alteration and optimisation of the actual sonophotocatalytic process can also drastically improve the wastewater treatment results. For instance, using gases (N_2_, Ar, O_2_) to purge the sonophotocatalytic reaction enhances the performance of the system by generating high-potential bubble formation sites. This then results in an increased rate of cavitation and greatly influences the amount of produced H_2_O_2_, which can actively participate in and contribute to the pollutant degradation process (Moradi et al., 2022).

The addition of oxidation agents (O_3_ and H_2_O_2_) has also been reported to enhance sonophotocatalytic efficiency (Joseph et al., 2009; Sathishkumar et al., 2016). The addition of H_2_O_2_ can cause the release of a large number of free and adsorbed radicals in the medium and it can act as an electron trap to suppress e^–^/h^+^ recombination (Moradi et al., 2022). For instance, Wei et al. (2021) observed an enhancement in the sonophotocatalytic performance of H_2_O_2_-assisted titania based system for degradation of Acid Orange 7 (Wei et al., 2021). Ozonation on the other hand can be utilised to enhance the production of reactive species either alone or in combination with ultrasound and or light irradiation (Kohantorabi et al., 2022).

Lastly, Fe-containing catalysts integrated with light and ultrasound improve the pollutant removal efficiency due to enhanced synergistic interaction in a process called sono-photo-Fenton catalysis (Moradi et al., 2020; Qi et al., 2020). The reaction of ferrous ions with *in situ* produced H_2_O_2_ during sonophotocatalytic reactions provides free additional radicals that facilitate the degradation of dissolved contaminants (Moradi et al., 2022). Unfortunately, the catalyst can suffer from poisoning during the oxidation process, due to the adsorption of reaction intermediates on its surface, thereby leading to a progressively inactive surface (Zhong et al., 2011). Segura et al. (2009) investigated the sono- and photo-Fenton processes separately as well as the combined systems (sono-photo-Fenton) in order to evaluate of possible beneficial effects on the use of coupled systems in the degradation of phenol. They observed enhanced activity for the sequentially-integrated sono–photo-Fenton process which resulted in a total phenol degradation and ca. 90% TOC reduction. They attributed the effective degradation of the sequential sono–photo-Fenton process to the benefits of ultrasound for the partial degradation of amenable aromatic compounds towards further oxidized by-products along with the fragmentation of catalyst to finer particles that produces more activity in photo-Fenton systems (Segura et al., 2009).

## 5 Challenges and future prospects

The majority of research conducted on sonophotocatalysis utilises UV-active materials (Taghizadeh and Abdollahi, 2011; Dinesh et al., 2015; Asgari et al., 2020), although some researchers have employed various modification methods to enhance the visible light absorption efficiency of the reported catalysts (Neppolian et al., 2011; Aziz et al., 2023). Research therefore needs to be conducted on the use of stable sonophotocatalysts with absorption in the Vis and NIR regions of the spectra so as to maximise utilisation of the sunlight. These include but are not limited to hematite and tungsten trioxide.

Many researchers have also reported on the anatase polymorph of TiO_2_ as the best catalyst for the photocatalytic treatment water pollutants relative to the rutile (Zhang et al., 2014; Theerthagiri et al., 2016). A comparative study on sonophotocatalysis using different TiO_2_ polymorphs at similar conditions therefore needs to be conducted to elucidate the best polymorph for sonophotocatalytic treatment.

Catalyst regeneration and reusability is another important aspect to wastewater treatment so as to prevent further contamination as a result of the catalyst (Argyle and Bartholomew, 2015; Taufik et al., 2016; Obaideen et al., 2022). Hence extensive research needs to be conducted on the sonophotocatalytic properties of retrievable materials including magnetic nanoparticles, films and fibers. The potential risks associated with the sonophotocatalytic process also need to be studied, including the possible generation of toxic byproducts and their potential release (and impact thereof) into the environment.

Research findings have depicted that the pollutant removal efficiency using sonophotocatalytic treatment is dependent on a range of parameters including pH, light source and power, ultrasound frequency and catalytic loading (Camacho-Alvarado et al., 2017; Yentür and Dükkancı, 2021). Moreover, a range of contaminants are likely to coexist in wastewater and not a single kind as most research tends to focus. This therefore suggests that interactions between different kinds of pollutants would complicate the sonophotocatalytic degradation process. Future studies are hence needed to evaluate the potential adverse effects of complex water systems with a range of pollutants, under different operating conditions. The application and optimization of sonophotocatalysis in environmental remediation also needs to be broadened to other resistant and toxic contaminants such as personal care products, oil, as well as other microbes like viruses and fungi.

Additionally, extensive research needs to be conducted on the influence of the sonophotocatalytic process on the stability of the catalysts at different conditions. This is a precaution to prevent the potential release (and impact thereof) of the sonophotocatalyst into the environment. Ideally, the process should not damage the catalyst’s physical and/or chemical structure, alter the morphology or disintegrate binary or ternary composites.

Lastly, although sonophotocatalytic treatment of water contaminants has been proven to be efficient, large scale up processes would not be economically viable without optimising the required facilities including suitable reactors as well as process operation and maintenance. Furthermore, the process requires electrical energy for generation of the ultrasounds which is a costly exercise.

## 6 Conclusion

Sonophotocatalysis has proven to be a highly efficient wastewater treatment process as it often shows higher removal rates and shorter reaction times when compared to its individual treatments i.e., sonocatalysis and photocatalysis. The ultrasound has a synergistic interaction with the light irradiation as it continuously cleans the catalytic surface and resolves problems related to catalyst opacity and porosity. Additionally, sonophotocatalysis eliminates the resistance in mass transfer between the liquid phase and the catalyst surface, eradicates catalyst fouling and promotes the generation of more free radicals (HO^•^, O_2_^•-^) for enhanced activity.

Features including the sonophotocatalysts (type, durability, stability, reusability) and treatment processes (irradiation intensity, scalability, reactor setup) are pivotal to the success of the sonophotocatalytic process. It can however be enhanced by sonophotocatalyst modification (doping, heterojunctions and dye photosensitisation) or process optimization (addition of gases, oxidants or ferrous agents).

Although the sonophotocatalytic treatment of dyes, pesticides, pharmaceuticals, heavy meatls and bacteria has been reported, research still needs to be conducted on the use of sonophotocatalysis in other pollutants like oils, personal care products, viruses and fungi, as well as complex water with a range of pollutants.

This review has thus shed light on the different water treatment applications that have been studied using sonophotocatalysis as well as the areas that need further exploration. It has also shed light on various aspects that still need to be considered and optimised to enhance the efficiency, real-life feasibility, largescale operation and commercial viability of sonophotocatalysis as a wastewater treatment technique.
